# Health monitoring and health indicators in Europe

**DOI:** 10.17886/RKI-GBE-2017-020.2

**Published:** 2017-03-15

**Authors:** Angela Fehr, Cornelia Lange, Judith Fuchs, Hannelore Neuhauser, Roma Schmitz

**Affiliations:** Robert Koch Institute, Department for Epidemiology and Health Monitoring, Berlin, Germany

**Keywords:** EUROPE, INDICATORS, HEALTH MONITORING, HEALTH REPORTING, CHRONIC DISEASES

## Abstract

Demographic change, new health threats and inequalities in health and healthcare provision in and between European Union (EU) member states pose a great challenge to European health care systems. Not only for these reasons does it make sense to collect comparable European health data. Such information provides insights on the distribution of risk and protection factors, the prevalence of chronic diseases and the levels of care provided in the member states and supports the planning and implementation of (health) policy measures. Since 2013, in the context of the European Health Interview Survey (EHIS), all EU member states are obliged to collect data on the health status, the provision of healthcare, health determinants and socio-economic conditions of their populations. In Germany, the EHIS is integrated into health monitoring at the Robert Koch Institute (RKI). The RKI is thus Germany’s interface to the European health monitoring presented here. European health monitoring relies on different indicator systems such as the European Core Health Indicators (ECHI), EU social indicators and the health indicators of the European Sustainable Development Strategy. These are based on administrative and survey data, which stem for example from the EHIS or the European Union Statistics on Income and Living Conditions (EU-SILC) survey. Comparative data analyses must take into account the differences between health care systems, socioeconomic conditions and the age structures of societies. Variances in the prevalence of allergies for example are also due to differences in the available diagnostic tools. Significant differences in the prevalence of hypertension in Europe (with a range of 20% for women and 17% for men) are also related to different levels of awareness of hypertension. Comparative analyses can support the planning of and provide information for policy measures, and enhance the sharing of experiences between EU member states. A forthcoming EU regulation aims to harmonise the content of and intervals between health and social statistical data collection. Moreover, plans exist to establish a European Research Infrastructure Consortium (ERIC), which is set to develop and institutionalise European health monitoring.

## 1. Background

**Info box 1: Treaty on the Functioning of the European Union, article 168 (Public Health)**

“1. A high level of human health protection shall be ensured in the definition and implementation of all Union policies and activities. Union action, which shall complement national policies, shall be directed towards improving public health, preventing physical and mental illness and diseases, and obviating sources of danger to physical and mental health. Such action shall cover the fight against the major health scourges by promoting research into their causes, their transmission and their prevention as well as health information and education, and monitoring, early warning of and combating serious cross-border threats to health. […]
2. The Union shall encourage cooperation between the Member States in the areas referred to in this Article and, if necessary, lend support to their action. […]
3. The Union and the Member States shall foster cooperation with third countries and the competent international organisations in the sphere of public health. […]
7. Union action shall respect the responsibilities of the Member States for the definition of their health policy and for the organisation and delivery of health services and medical care [2].“

‘Health’ has gradually developed as a European policy field. During the 1950s, the predecessor to the European Commission provided support to member states in the field of occupational health and safety for coal and steel industry workers. Today, European health policy covers fields such as disease prevention and control, provision of medicines and health research. Yet, for European citizens, the main responsibility for health policy and health care still remains at member state level. The EU supports, coordinates or supplements measures that are adopted by the member states (article 6 of the Treaty on the Functioning of the European Union, TFEU) [1]. Article 168 TFEU defines the framework and goals of this supplementing competency of the EU and emphasises the importance of health for all EU policy fields (see Info box 1).

To complement this, and in the face of current challenges to health care systems – demographic change, migration, the financial crisis, new health threats (such as pandemics, bio-terrorism and climate change) and advances in medical technologies – the EU developed its health strategy ‘Together for Health’. The strategy complements the Europe 2020 strategy and its focus on increasing productivity and competitiveness in the EU. Within this context, investments into health care are to help reduce health inequalities and combat social exclusion. Overarching values such as universality, access to good quality care, equity and solidarity are to guide the systems for the provision of healthcare in the EU and take into account the gender perspective [3].

For European health care systems to react adequately to the above-mentioned challenges and develop strategies to improve health will require regular data on the development of living conditions, health status and behaviour, and healthcare levels in Europe. The European statistics office (Eurostat), the Organisation for Economic Co-operation and Development (OECD) and the World Health Organisation (WHO regional office for Europe) all provide and publish data on health in Europe at regular intervals. Every two years, the OECD, in cooperation with the European Commission, publishes its Health at a Glance report [4] and every three years the WHO’s regional office for Europe publishes its European health report [5].

Indicators that aim for the greatest comparability between countries and allow for quantified assessments of the health status, health behaviour and determinants for health and well-being are the basis of these reports. In the following, this article focuses on EU indicator systems and their sources of data. As an example, it provides comparative analyses for selected chronic diseases from the second wave of the European Health Interview Survey (EHIS-2). To conclude, the article discusses the perspectives and limitations of European health monitoring.

## 2. EU health indicator (systems)

As standardised measures, indicators can help reveal processes, results or changes at a certain moment in time or over a period of time [6-8]. Standardised data collection on the basis of European health indicators is of fundamental importance for the development of national and European research and health policies [9]. This helps identify and close gaps in the data and contributes towards data-based objectivity and scientific orientation of health policy discussions. EU indicator systems relevant to health include the European Core Health Indicators (ECHI), European social indicators and the public health indicators of the European Sustainable Development Strategy, which are described in more detail below. Numerous links exist between these systems and with sets of indicators on specific topics such as accidents or child and youth health.

### 2.1 European Core Health Indicators (ECHI)

At the end of the 1990s, the EU decided to establish “Community Health Indicators” [10]. Between 1998 and 2012, four projects (ECHI-1, ECHI-2, ECHIM, JA ECHIM) led to the development of the ECHI shortlist with 88 indicators (European Core Health Indicators). ECHI indicators rely on a large number of data sources. To ensure the comparability of data, each indicator has a defined preferred data source (such as Eurostat) and a preferred type of data (such as survey data) [11]. Section 3 (“Data sources for EU health indicators”) describes a number of data sources for EU health indicators in detail. Data collected on the basis of the ECHI indicators can be accessed, downloaded or visualised in aggregated form on the EU’s publicly accessible ECHI Data Tool [12].

The declared aim was not to select indicators based solely on data availability [13]. Correspondingly, the ECHI shortlist is divided into three sections that reflect the different degrees of implementation readiness of specific indicators. Data from defined international data sources from the preferred type of data is available for 67 indicators (implementation section). A further 14 indicators (work-in-progress section) have been sufficiently developed both at the conceptual and methodological level, yet final obstacles to their Europe-wide implementation remain. The third group (development section) comprises topics of issues that, although relevant to health policy, still require discussion before they can be developed conceptually and methodologically into indicators [11]. The inclusion of new indicators or the transfer of indicators between sections is based on consensually decided criteria. Documentation forms are available for all indicators and structured remarks on their comparability are provided for most of them. The majority of implemented indicators can be stratified according to age, gender, education and region where this is considered useful.

The implementation of indicators in the health information systems of member states began in 2005. Numerous EU countries have already implemented ECHI indicators in national health monitoring. Federal health reporting data in Germany is also provided according to the definitions in the ECHI shortlist.

**Fig. 1:**
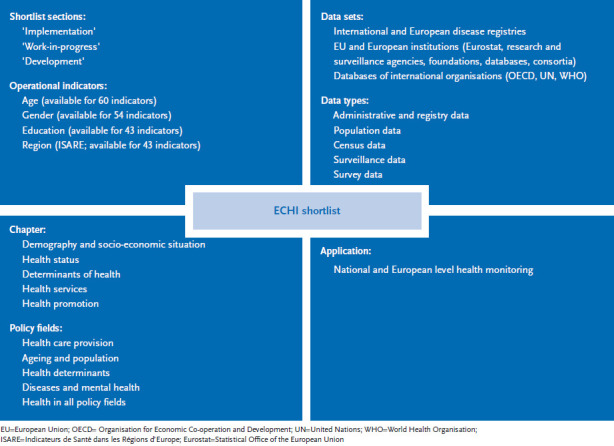
ECHI shortlist structure, data sets, application Source: own chart based on [16, 17]

The further development of the ECHI shortlist is part of the EU-funded BRIDGE health project (BRidging Information and Data Generation for Evidence-based health policy and research). Generally, the goal is to keep the list of indicators as stable as possible. For different reasons, however, it may become necessary to update indicators. Such reasons include changes to EU health surveys, new relevant public health concerns or changes to health policy concepts. A good example here are shifts in the conceptual approaches toward disability. Whereas in the past questions used to focus on a person’s disabilities, they today ask about the barriers people with disabilities face for their full participation in society [14]. Figure 1 illustrates the policy fields, sections and data of ECHI indicators. Cancer incidence, self-reported prevalence of asthma, diabetes, depression and chronic obstructive pulmonary disease (COPD) are all among the non-communicable diseases ECHI indicators currently monitor. Indicators for acute myocardial infarction (AMI), stroke and register-based prevalences of asthma, COPD, diabetes, dementia and depression are still under development. Cause of death statistics are a further source of information on disease in Europe. The corresponding indicator in the ECHI shortlist (indicator 13) includes 26 ICD-10 causes of death. Cause of death statistics, however, offer no information on disease incidence and prevalence. Future Europe-wide diagnosis-specific morbidity statistics should close this gap also in the data on chronic disease. Eurostat in collaboration with EU member states is currently conducting corresponding pilot studies [15].

**Info box 2: EU social indicators**

EU social indicators are an instrument that was developed in the context of the strategy to promote growth, a dynamic economy and social cohesion in the EU (the Lisbon Strategy), adopted by the European Council in 2000. In social policy, the EU has shared competence and coordinating capacity (articles 4 and 5 TFEU) [1]. To implement the goals of the Lisbon Strategy, Europe developed the open method of coordination (OMC). The method was ‘launched […] as voluntary self-evaluating process, based on common objectives, […]’ [18]. In the area of health, social indicators are to allow for self-evaluation, comparison and benchmarking in healthcare and long-term care provision. Since 2005, social indicators have been the basis for the European Survey on Income and Living Conditions, EU-SILC. It comprises the Minimum European Health Module (MEHM), consisting of seven variables [19].

### 2.2 EU social indicators relevant to health

**Fig. 2:**
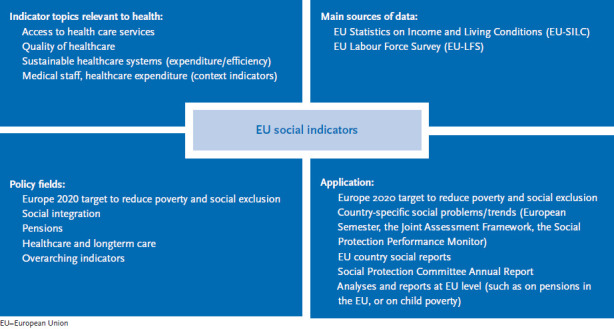
EU social indicators, health issues, data sources, application Source: own chart based on [20]

In addition to the Core Health Indicators, the system of EU social indicators also reflects questions relevant to health. They are embedded in the context of EU social policy and focus on healthcare provision and long-term care (see Info box 2; Figure 2).

In 2010, the Europe 2020 strategy succeeded the Lisbon Strategy and provided a greater focus on questions of health in Europe. The 2013 EU Annual Growth Survey emphasised the need to analyse how the healthcare systems of member states can manage the twofold challenge of ensuring access to high quality healthcare services whilst keeping the costs at manageable levels in the long term [18, 21]. Moreover, the strategy in particular recognised the need to reduce inequalities between member states, regions and socio-economic groups [22]. Adjusted indicators to reflect this fact are being developed at EU level with the participation of member states. These efforts are based on the Joint Assessment Framework (JAF) method [23]. JAF health indicators aim to enhance the evidence basis of EU health policy activities and recommendations to member states [24]. The set of indicators currently being developed includes EU social indicators and indicators from the ECHI shortlist. Among these are outcome indicators such as life expectancy, healthy life years, self-perceived health, infant mortality and causes of death. Further indicators shed light on the accessibility and quality of healthcare services such as unmet healthcare needs, vaccination coverage, screening, avoidable hospital admissions as well as on health determinants (tobacco and alcohol consumption, physical activity, obesity, and fruit and vegetable consumption). Additional (called contextual) indicators provide information on healthcare expenditure as well as on sociodemographic factors [25]. The set of indicators differentiates between EU indicators, national (NAT) indicators as well as contextual indicators. Only EU indicators are applicable for comparisons between member states.

### 2.3 Sustainability indicators

In 2001, the European Council adopted the EU Sustainable Development Strategy, which was renewed in 2006. Public health is one of the ten issues the EU has identified as key challenges to sustainable development and for which it states targets, operational objectives and actions. Progress is measured based on around 130 indicators [26].

Focuses for the complex of public health are the promotion of good public health on equal conditions for all citizens and improved protection against health threats. Indicators such as life expectancy, healthy life years and chronic disease mortality rates are important as is data on the production of toxic chemicals, air or noise pollution and accidents at work. Further indicators include unmet healthcare needs, long-term illness or health conditions [27]. The sustainability indicators are also based on administrative health data as well as on data from social and economic monitoring. Every two years, a monitoring report is published; the most recent one was published in September 2015 [28].

Some are stand-alone health surveys, whilst others contain health-related modules or variables within a larger overarching complex of issues. Table 1 presents an overview of European health surveys and indicators. The following section then focuses on the most important data sources for health indicators.

## 3. Data sources for EU health indicators

Data availability is addressed early in the process of conceptualizing and developing European and international sets of indicators. This ensures that meaningful and comparable data will be available for all countries involved. Individual indicators are taken from different data sources and data owners. Among these are the EU’s statistical office (Eurostat), the World Health Organisation’s European ‘Health for all’ database (HFA-DB) [31], the database of the Organisation for Economic Co-operation and Development (OECD) [32] as well as international reporting systems for specific issues, for example on tobacco, alcohol and drug consumption, accidents or environmental monitoring. The types of data include official statistics, interview and administrative data. Eurostat for example provides demographic data, population projections, data on mortality, life expectancy, fertility, migration and citizenship based on official statistics for all EU member states. The European Union Labour Force Survey (EU LFS), conducted by all EU member states, two EU candidate countries and three countries of the European Free Trade Association, provides important data on the labour market and employment.

Important survey data for health indicators at European level stems from the European Survey on Income and Living Conditions (EU-SILC) and from the European Health Interview Survey (EHIS). One quarter of implemented ECHI indicators is collected in the context of EHIS [33].

### 3.1 European Survey on Income and Living Conditions (EU-SILC)

EU-SILC is the EU’s source of reference for comparable statistical data on income distribution and social inclusion at European level. The survey provides two types of data on an annual basis for the 28 EU members, Iceland, Norway, Switzerland and Turkey:

► Cross-sectional data at a particular moment or time-span on income, poverty, social exclusion and further living conditions;
► Longitudinal data on changes over time at individual level, periodically surveyed during a specific period, usually four years.

The fundament of EU-SILC is a shared “common framework” and not a unitary questionnaire or joint data collection. This framework consists of a harmonised list of primary (annual) and secondary (collected at least every four years) target variables, which countries commit to transmitting to Eurostat. It also contains overarching guidelines and procedures, shared concepts (household and income) and classifications to ensure the greatest possible comparability of the data provided [34]. EU-SILC was initially implemented throughout Europe with framework regulation (EC) 1177/2003 in 2005. In the 2014 wave, the sample in Germany covered 12,744 households and 22,695 people aged at least 16.

The overarching goal and primary purpose of EU-SILC is to provide European and national level social policy with a well-grounded basis for decisions. EU-SILC provides the basis for monetary and non-monetary social indicators. An important social indicator provided by EU-SILC is the at-risk-of-poverty rate [35]. Included in EU-SILC is the Minimum European Health Module [36], which surveys people’s self-perceived health, the presence of chronic diseases and of long-standing limitations in daily activities (Global Activity Limitation Indicator, GALI). This module is also part of the EHIS. Further health-related surveys could in future also use parts of this module. GALI data is used to calculate the Healthy Life Years (HLY) indicator (also called disability-free life expectancy). In the Lisbon Strategy, the indicator belonged to the group of core European structural indicators. Today, HLY is one of two public health headline indicators in the EU’s Sustainable Development Strategy. EU-SILC also includes questions on why people did not go to see a doctor or a dentist, even though this would have been medically necessary [35].

### 3.2 European Health Interview Survey (EHIS)

A 2008 EU regulation [37] called on member states to supply health statistics in the following domains: health status and health determinants, causes of death, health care, accidents at work, occupational diseases and other work-related health problems and illnesses. The European Health Interview Survey (EHIS), which is to be conducted every five years, is to serve as the primary basis for data on health status and health determinants. EHIS is an interview survey which relies on the self-assessment of participants. Unlike in examination surveys, no measurements are collected. The first wave of EHIS was conducted between 2006 and 2009. Participation in EHIS 1 was not mandatory and 17 member states took part [38]. Germany partly included the EHIS instrument in the Robert Koch Institute’s (RKI) German Health Update (GEDA 2010) survey and supplied Eurostat with results for certain health indicators.

After an intensive process of evaluation of the first wave, development of the second and mandatory wave of EHIS began. The modules on mental health, physical activity as well as on alcohol consumption underwent revision. After two further years of work and discussion to develop a binding catalogue of questions, an EU regulation was adopted in February 2013 that defined the EHIS variables, reference year and population as well as the required reference meta data [39].

EHIS consists of four modules on health status (self-assessed), health care, health determinants and background demographic and socio-economic status variables. To ensure the broadest possible comparability of the data received from member states, Eurostat also developed a detailed manual, which also includes a model questionnaire [40]. The German language questionnaire is provided as a supplement to this edition. Data should be collected during the reference years 2013, 2014 or 2015. In Germany, EHIS 2 was integrated into GEDA 2014/2015. Beyond the EHIS questions, this survey asked further nationally relevant questions to continue earlier time series and/or collect information on specific public health aspects.

The EU regulation on EHIS lists all the variables for the survey including the answer categories. Countries freely choose their method of data collection and define the details of how the survey is conducted. EHIS can be conducted as a stand-alone survey or EHIS questions can be integrated into a national health survey, as was the case in Germany. A further article in this edition describes the methodology applied in GEDA 2014/15-EHIS. EHIS also defines the target population (individuals aged 15 and over living in private households residing in the territory of the Member State at the time of the data collection) and the sample size each country needs to achieve (in total around 195,000 participants in EU member states). To assist data processing and data quality assurance, Eurostat provides detailed documents and electronic tools. Eurostat controls the microdata received from member countries for plausibility and completeness. Each country is required to fill out a detailed quality report based on pre-defined criteria, which details information on the methodological approach chosen by each country.

Based on this quality assured data, Eurostat calculates indicators, which the office usually stratifies according to age, gender and education levels. These are available on the Eurostat website [41].

## 4. Chronic diseases as an example of EHIS results

EHIS delivers standardised and periodically collected core indicators for health monitoring from all countries at European level. EHIS thereby mainly contributes indicators on health status and health determinants for which no broad and comparable data basis was so far available from the various countries. In addition to the European indicator systems (mentioned above) and the possibility to access indicators through the Eurostat database [41], the indicators will be used by the national health reporting systems. In Germany, the Journal of Health Monitoring publishes Fact sheets on specific indicators and updates these over time taking into account newly collected data. The Fact sheets in this issue discuss indicators for selected chronic diseases with particular public health relevance: coronary heart disease, stroke, hypertension, diabetes and allergies. Future issue of the Journal of Health Monitoring will focus on further indicators such as arthrosis, asthma and other chronic lower respiratory infections (chronic bronchitis, chronic obstructive pulmonary disease and emphysema), which are presented in brief below.

Overall, EHIS collected information on the occurrence of fifteen frequent chronic diseases and health conditions through the following question: ‘During the past 12 months, have you had any of the following diseases or conditions?’ Very clearly, therefore, the survey asked for self-perceived illnesses and not self-reported medical diagnoses, diagnostic criteria based on objective measurements or other disease-specific information such as taking certain medications. 12 months was chosen as a reference period because from a health policy point of view information on the current prevalence of a disease in the population is of higher value than its lifetime prevalence. Moreover, this establishes a direct reference to ECHI indicators 21a (self-reported diabetes), 23a (self-reported depression), 26a (self-reported asthma), 27a (self-reported COPD) and 43 (self-reported high blood pressure) that are all based on twelve-month prevalence. Table 2 shows the fifteen diseases considered in the EHIS 2 survey.

There are, however, inherent limitations of international comparisons of EHIS indicators, just like with other international indicator systems. On the one hand, this is due to the indicators themselves. On the other hand, in spite of all efforts to make data collection comparable, numerous differences between countries exist in life expectancy, age structure, the organisation and performance of the healthcare system, factors concerning the socio-economic context and cultural factors; these have to be taken into account when interpreting results [42]. Consequently, simple benchmarking, e.g. a one-dimensional perspective on the ranking of individual indicators, makes little sense, even more so since Eurostat so far does not provide age-standardised prevalence data.

**Fig. 3:**
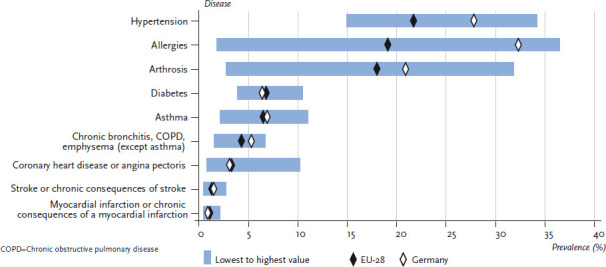
12-month prevalence of selected diseases among women in Germany compared to the EU-28 mean Source: EHIS 2014/15

**Fig. 4:**
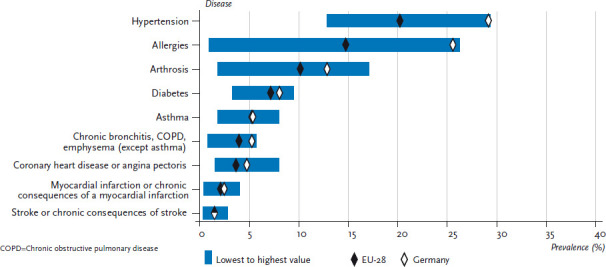
12-month prevalence of selected diseases among men in Germany compared to the EU-28 mean Source: EHIS 2014/15

Figures 3 and 4 therefore present the prevalence of the above-mentioned selected chronic diseases according to gender in Germany in comparison with the EU average including the range between the lowest and highest reported values. This analysis did not include indicators referring to symptoms, to low-prevalence conditions or to mental health.

Whilst prevalence of individual diseases varies considerably across the EU, patterns in women and men are quite similar. Country-specific prevalences of asthma and other chronic lower respiratory infections, myocardial infarction, stroke, coronary heart disease and diabetes vary by less than ten absolute percentage points. Variation in prevalence is much higher in hypertension (20% in women, 17% in men), arthrosis (29% in women, 15% in men) and allergies (35% in women, 25% in men).

**Fig. 5:**
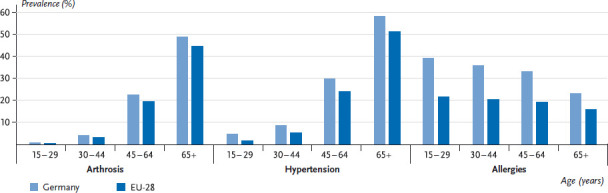
12-month prevalence of arthrosis, hypertension and allergies among women, Germany and EU-28 according to age Source: EHIS 2014/15

**Fig. 6:**
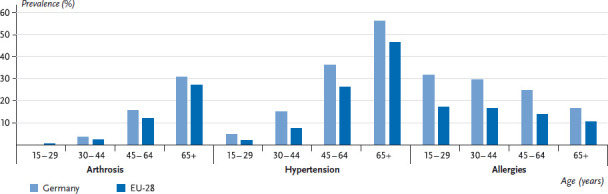
12-month prevalence of arthrosis, hypertension and allergies among men, Germany and EU-28 according to age Source: EHIS 2014/15

Figures 5 and 6 focus on the three chronic diseases with the largest cross-country differences and show prevalences according to age and gender for Germany compared to the EU average. The results confirm for all three diseases that prevalences are high in Germany as reported from various previous health surveys [43-46]. For both genders, the estimated prevalence of arthrosis and hypertension in Germany is slightly higher than the EU average and considerably higher for allergies. Also in line with earlier data from Germany, a clear correlation was found between age and prevalence of these diseases. A consistent age-related increase in the prevalence of arthrosis and hypertension, and an age-related decrease in the prevalence of allergies were found for women and men [43-46]. The Fact sheets on allergies and hypertension in this issue also point to this fact.

###  

#### Prevalence of arthrosis

In Germany slightly more respondents reported suffering from arthrosis than the EU average across all age groups. The range of prevalence rates among men was between 1.8% and 17.1% (10.1% across the EU, 12.8% in Germany), and between 2.7% and 31.8% among women (18.0% across the EU, 20.9% in Germany). Globally, arthrosis is the most common disease of the joints and mainly affects people in the second half of life. The percentage of those affected increases with age, and women are affected more frequently than men [47]. In contrast to diseases with early detection programs like diabetes (medical check-up examinations measuring glucose levels), arthrosis is usually not detected until joints (frequently the hips, knees, arms and hands, shoulders, ankles or feet) are affected. Only medical specialists using imaging techniques can explain to what degree these symptoms are related to joint degeneration. Both people’s perception of joint problems and also their diagnosis differ between countries [48, 49]. Moreover, when asked about arthrosis, people will often include all kinds of problems with their joints, without having a physician-diagnosed arthrosis. Obesity, joint injuries at a younger age and hard physical work are considered important risk factors for arthrosis and these factors are spread unevenly in the EU. Individually and in combination, these factors contribute to the differences in prevalence.

#### Prevalence of hypertension

Large differences in the prevalence of hypertension across Europe have also been reported previously from examination surveys [50]. Since not only the prevalence of hypertension, but also people’s awareness of hypertension, varies between European countries, the differences in prevalence within the EU revealed by the EHIS indicator ‘self-reported hypertension in the past 12 months’ are hardly surprising. For men, prevalences range between 12.9% and 29.4% (EU-wide 20.2%, Germany 29.2%) and for women between 14.9% and 34.2% (EU-wide 21.7%, Germany 27.8%). As other analyses have shown, the higher prevalence of self-reported hypertension among women is due almost entirely to their greater awareness of hypertension. When diagnosed in examination surveys, however, which also account for undetected cases of hypertension, the prevalence of the condition is higher among men in most populations [51]. EHIS prevalences of self-reported hypertension in some countries are slightly lower, in other countries considerably lower than prevalence of known hypertension reported from country-specific examination surveys [50]. One reason could be that EHIS questionnaires do not grant that participants who take medicines and whose hypertension is therefore controlled identify themselves as having hypertension. This point should be discussed in a review and further development of EHIS indicators. For the corresponding indicator in the ECHI shortlist (indicator 43), this point was annotated to be considered during a later review [11].

#### Prevalence of allergies

With regard to allergies, relatively large differences in prevalences between European countries and globally have been known for quite some time. The EU average for allergy prevalence based on self-reports is 16.9%. The EU-wide range of allergy prevalences varies from 1.4% to 31.6%. In Germany, the prevalence is 29.0%. On the one hand, the large range could express that the risk for allergies increases the more sterile an environment is in which a person grows up and the fewer a person’s immune system has opportunities to deal with pathogens and allergens (Hygiene hypothesis [52]). Cross-sectional studies have revealed that children who grow up on farms are less likely to develop allergies. On the other hand, the indicator allergy probably covers a number of diseases, presumably also pseudo allergies with a broad spectrum of symptoms and degrees of severity such as food intolerances. The degree of awareness and practical applicability of allergy diagnoses and testing, as well as of specific immunotherapy (desensitisation), vary between countries [53].

## 5. Discussion and outlook

Chronic diseases are among the great challenges for which not only the EU member states have to prepare. International and European comparisons on the basis of shared indicators can broaden the perspective of national health reporting and reveal the need for (health) policy action. They can also provide a basis for EU member states to share their experiences. Still, methodological and structural constraints need to be considered which hamper European and international comparisons. To illustrate, Eurostat, WHO or OECD data, which is used in international comparisons, or data from specific surveys may vary from the data used at national level for the same matter. Moreover, national reports may show different values than international reports for certain indicators (such as the mortality rate) if data has been standardised for age for an international comparison. Different statistical procedures can also lead to differences between national and international results. As concerns the ECHI, remarks on comparability have been developed for those indicators which are not based on EHIS data, but for which data from other international sources is available. They explain the factors that need to be taken into account in cross-country comparisons or that have led to the interruption of time series in data collection. In addition, contextual factors have to be considered in the interpretation of comparisons between countries and regions. These include the heterogeneity of healthcare systems in Europe, the differences in the availability of relevant data (information inequality) as well as cultural differences in understanding and dealing with health and illness. The critical appraisal of the EHIS data presented here exemplifies this fact.

Numerous projects at European level promote the further development of social and health indicators, aiming to improve the availability and comparability of data. To illustrate, so far, social statistics surveys in Europe were implemented on the basis of separate regulations. In the context of modernising European social statistics, they shall become integrated into the single framework of the Integrated European Social Statistics (IESS) regulation. This is to ensure that there is a stable data basis to strengthen the EU’s social goals. The process began in 2014, aims for adoption between 2017 and 2018, and implementation in 2019.

In the same vein, namely to provide better and comparable information on the health of Europeans which would allow the EU to evidence-base its health policy activities, plans are progressing towards the establishment of a European Health Information System. The preferred structure is that of a European Research Infrastructure Consortium on Health Information for Research and Evidence-based Policy (HIREP-ERIC). An ERIC is an international institution borne by its participating countries and is open not only to members of the EU but also to third countries and specialised agencies. Significant work to this end is done in the EU-funded BRIDGE Health project (2015-2017, http://www.bridge-health.eu). The further development of ECHI indicators and their increasing implementation in the systems of EU member states, EU candidate countries as well as the European Free Trade Association (EFTA) countries will be among the most important activities. A sustainable EU infrastructure for health information creates the necessary framework, con tinuity over time and platform for exchanges between experts. The overarching goal remains to improve the availability and comparability of data relevant to health, strengthen the evidence basis for policy and promote the exchange of knowledge in Europe.

## Key statements

- Comparable health data is needed to support the planning and implementation of health policy measures in Europe.
- The key European indicator systems are the European Core Health Indicators (ECHI), EU social indicators and the health indicators of the European Sustainable Development Strategy.
- Since 2013, all EU member states are obliged to collect health data in the context of the European Health Interview Survey (EHIS).
- Great differences in prevalence of arthrosis, high blood pressure and allergies can be observed in Europe.
- Data analysis must take into account the differences between health care systems, socio-economic conditions and/or age structures.

## Figures and Tables

**Table 1:** Data sets for European health statistics Source: [27, 29]

	Focus	Health indicators
**EHIS** (European health interview survey)*	Health	Demography and socioeconomic situation, health status, health determinants and measures by the healthcare system
**EU-SILC** (EU statistics on income and living conditions)*	Income and living conditions	Health status and utilisation of the healthcare system (minimum European health module: MEHM, seven variables)
**EU LFS** (EU Labour Force Survey)*	Labour market conditions and trends	Employment of disabled people (2002, 2011); accidents at work and other work-related health problems (1999, 2007, 2013) (ad-hoc modules)
OECD/Eurostat/WHO in the **System of Health Accounts** (SHA)	Health expenditure	Including costs of investments in the health sector, income from programmes to finance healthcare, contributions to cover the costs of healthcare goods and services, health expenditure based on essential characteristics of service recipients
OECD/Eurostat/WHO Europe on **non-monetary health care benefits**	Health care system resources and measures	Hospital discharges, hospital stays, medical procedures, selected preventive measures and consultations
**Causes of death**	Cause of death statistics	ICD causes of death
**Accidents at work**	Statistics on accidents at work	Non-fatal/fatal accidents at work
**Diagnosis-specific morbidity** (pilot phase, regular data collection possibly from 2020)	Incidence and prevalence of diseases	Diseases (list currently under development)

* planned future implementation under the Framework regulation for the production of European statistics – Integrated European Social Statistics, IESS [30]

OECD=Organisation for Economic Co-operation and Development; Eurostat=Statistical office of the European Union; WHO=World Health Organisation; ICD=International classification of diseases

**Table 2:** Chronic diseases EHIS-2 Source: GEDA 2014/15-EHIS, German questionnaire (see supplement of the German issue)

Chronic diseases considered in the EHIS 2 survey
Asthma (allergic asthma included)
Chronic bronchitis, chronic obstructive pulmonary disease, emphysema
Myocardial infarction or chronic consequences of myocardial infarction
Coronary heart disease or angina pectoris
Hypertension
Stroke or chronic consequences of stroke
Arthrosis (arthritis excluded)
Low back pain or other chronic back symptoms
Neck pain or other chronic neck symptoms
Diabetes
Allergy, such as rhinitis, hay fever, eye inflammation, dermatitis, food allergy or other (allergic asthma excluded)
Cirrhosis of the liver
Urinary incontinence, bladder control problems
Chronic kidney disease or renal failure
Depression
