# Corrigendum: Health monitoring and health indicators in Europe

**DOI:** 10.25646/6791

**Published:** 2018-10-18

**Authors:** Angela Fehr, Cornelia Lange, Judith Fuchs, Hannelore Neuhauser, Roma Schmitz

Fehr A, Lange C, Fuchs J et al. (2017)

Journal of Health Monitoring 2(1): 3-21.

DOI 10.17886/RKI-GBE-2017-020

In the original article, in the section ‘Prevalence of allergies’ only the prevalence for women was reported instead of the overall prevalence for women and men. In the current version, the sentence has been corrected as follows: ‘The EU average for allergy prevalence based on self-reports is 16.9%. The EU-wide range of allergy prevalences varies from 1.4% to 31.6%. In Germany, the prevalence is 29.0%.’

The corrected version of the article is available at DOI 10.17886/RKI-GBE-2017-020.2

